# Non-Coding RNA in Pancreas and β-Cell Development

**DOI:** 10.3390/ncrna4040041

**Published:** 2018-12-13

**Authors:** Wilson K. M. Wong, Anja E. Sørensen, Mugdha V. Joglekar, Anand A. Hardikar, Louise T. Dalgaard

**Affiliations:** 1 NHMRC Clinical Trials Center, University of Sydney, Camperdown, Sydney, NSW 2050, Australia; wilson.wong@ctc.usyd.edu.au (W.K.M.W.); mugdha.joglekar@ctc.usyd.edu.au (M.V.J.); anand.hardikar@ctc.usyd.edu.au (A.A.H.); 2 Department of Science and Environment, Roskilde University, DK-4000 Roskilde, Denmark; elaine@ruc.dk

**Keywords:** non-coding RNAs, long non-coding RNAs, long intergenic non-coding RNAs, microRNA, small nucleolar RNAs, piwi associated RNAs, circular RNAs, β-cell, α-cell, islets of Langerhans, pancreas, fetal development

## Abstract

In this review, we provide an overview of the current knowledge on the role of different classes of non-coding RNAs for islet and β-cell development, maturation and function. MicroRNAs (miRNAs), a prominent class of small RNAs, have been investigated for more than two decades and patterns of the roles of different miRNAs in pancreatic fetal development, islet and β-cell maturation and function are now emerging. Specific miRNAs are dynamically regulated throughout the period of pancreas development, during islet and β-cell differentiation as well as in the perinatal period, where a burst of β-cell replication takes place. The role of long non-coding RNAs (lncRNA) in islet and β-cells is less investigated than for miRNAs, but knowledge is increasing rapidly. The advent of ultra-deep RNA sequencing has enabled the identification of highly islet- or β-cell-selective lncRNA transcripts expressed at low levels. Their roles in islet cells are currently only characterized for a few of these lncRNAs, and these are often associated with β-cell super-enhancers and regulate neighboring gene activity. Moreover, ncRNAs present in imprinted regions are involved in pancreas development and β-cell function. Altogether, these observations support significant and important actions of ncRNAs in β-cell development and function.

## 1. Introduction to Pancreas and Islet Cell Development

The pancreas is a unique organ in our body consisting of two major compartments: The exocrine pancreas and the endocrine pancreas. The exocrine pancreas is composed of acinar cells, which produce digestive enzymes; and an intricate network of pancreatic ducts that carry these enzymes to the intestine in order to aid in digestion of the ingested food. The endocrine pancreas, also known as the “Islets of Langerhans”, is interspersed within the exocrine portion of the pancreas. Islets constitute a group of cells and can be considered as mini-organs themselves owing to the varied nature and function of islet cells. Each islet is composed of hormone-secreting as well as vascular endothelial and neural cells. The five hormone-producing cells within the islets secrete glucagon (α-cells), insulin (β-cells), somatostatin (δ-cells), pancreatic polypeptide (PP-cells) and ghrelin (ε-cells), that are necessary/important in glucose homeostasis and metabolism. 

**Figure 1:**
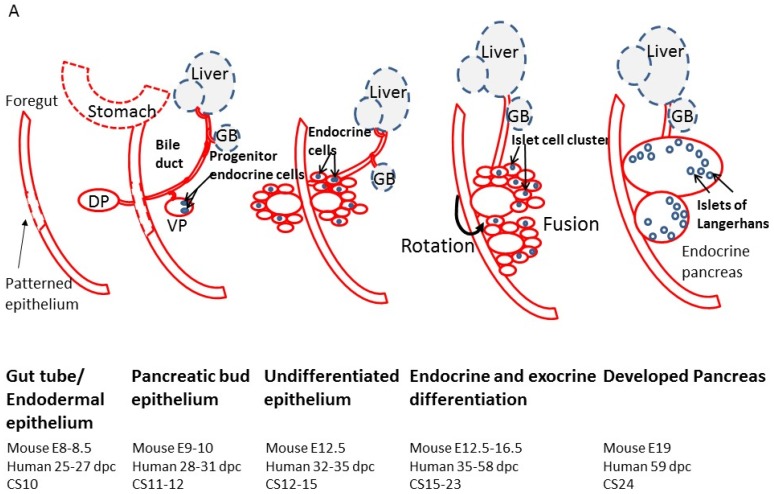

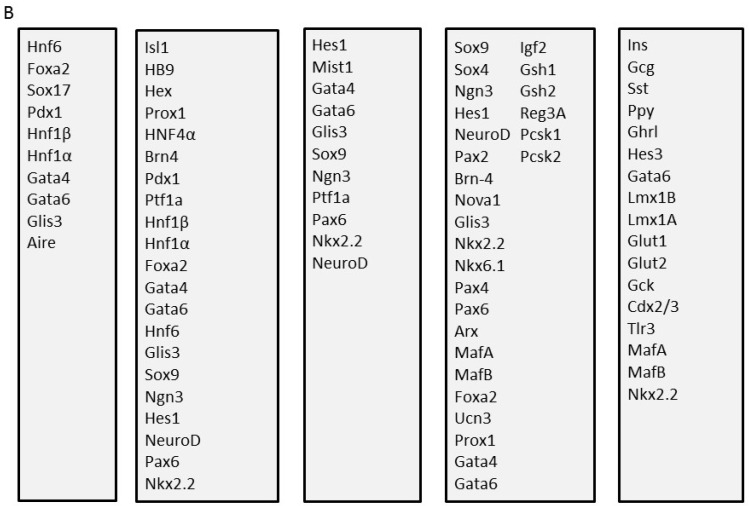
(**A**) Diagram of the major morphogenic events during islet development. (**B**) A cascade of different transcription factors, hormones and cell specific markers are expressed within different stages of pancreatic development that are responsible for the morphogenic events leading to islet formation and cellular differentiation. The diagram was inspired by [6,13]. DP, Dorsal pancreatic bud; VP, Ventral pancreatic bud; GB, Gall bladder; dpc, days post conception; CS, Cambridge stage.

Pancreas development is an orchestrated process that involves highly regulated expression of several transcription factors at the spatio-temporal level. This phenomenon is well studied in mouse models; however, limited availability of human fetal specimen has restricted detailed studies on human pancreas development. Nonetheless, there is a major overlap between these transcription factors between humans and rodents [1,2,3,4,5,6,7,8] (Figure 1). The pancreas is an endocrine organ that develops from the primitive gut tube in the form of a dorsal bud and a ventral bud (mouse embryonic (E) day 9–10; human 28–31 days post coitum (dpc)) arising at the junction of foregut and midgut [9] (Figure 1). The two mesodermal structures; notochord and the dorsal aorta signal to the prospective pancreatic epithelium, activating a cascade of downstream transcription factors and signaling molecules, that induce the initial expression of Pancreas and duodenal homeobox gene 1 (Pdx1, also known as insulin promoter factor 1 (IPF1)) [10,11,12]. In the undifferentiated epithelium (mouse E12.5; human 32–35 dpc), single endocrine cells are scattered within the ventral and dorsal pancreatic buds. Between mouse E12.5 to E16.5 (human 35–58 dpc) the pancreatic buds begin to undergo extensive branching morphogenesis and commit to endocrine or exocrine cell lineages, which further proliferate, expand and differentiate. Both pancreatic buds continue to grow independently until the secondary transition occurs, where the stomach and duodenum rotate and bring the dorsal and ventral buds in close proximity. Both buds then fuse by mouse E15–16.5 (human 53–58 dpc) and now constitute the definitive pancreas (Figure 1). Eventually, the dorsal bud becomes the tail, body and accessory pancreatic duct of the definitive pancreas, while the ventral bud forms the pancreas head and main pancreatic duct. By this time, the pancreatic endocrine precursors migrate in response to various paracrine as well as autocrine signals and cluster together to form islet-like structures.

The mesenchyme surrounding the pancreatic epithelium secretes several growth/differentiation factors that promote the maturation of endocrine progenitor cells. These growth/differentiation factors, along with the interactions of progenitor cells with neighboring cells as well as with the matrix provide cues for final stages of maturation into functional hormone-producing islets of Langerhans. 

Pancreas organogenesis follows an intricate pattern of endocrine gene expression. Various transcription factors (such as Pdx1, Ngn3, NeuroD1, NKX2.2, Pax6, Isl1) reported at different stages of pancreas development are shown in Figure 1 and reviewed in detail elsewhere [1,2]. These transcription factors have different expression patterns, with some are seen for a very short timeframe, some continue longer while some others, such as Pdx1, are seen from early development and also in the adult β-cells. The five different hormone-producing cells start appearing as single cells at different stages with glucagon-producing cells being detected at E9.5; insulin-producing cells detectable by E10.5; somatostatin-producing cells at E14.5. Ghrelin-producing ε cells and pancreatic polypeptide-secreting PP cells develop in later stages as close to the day of birth in mice [9]. The careful orchestration of multiple pancreatic transcription factors, thus drives the differentiation of committed islet progenitor cells, to form the islet micro-organ. It is now emerging that other molecules, mainly the non-coding RNAs (ncRNAs), also play an important regulatory role in pancreas and islet-cell development. 

## 2. A Primer on Short and Long Non-Coding RNAs

### 2.1. Categorization of Non-Coding RNAs

**Figure 2:**
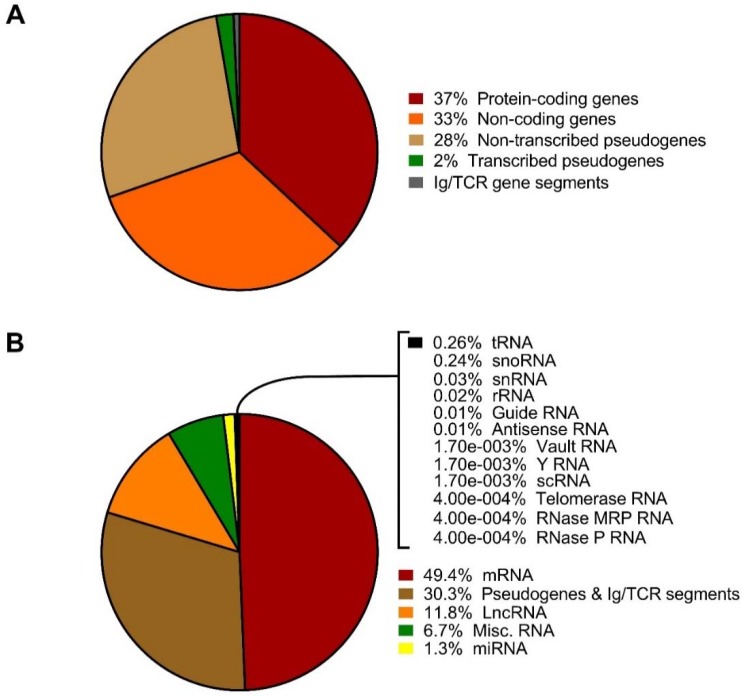
Pie charts showing the percentage of (**A**) the different genes and (**B**) different RNA types from the human reference genome (GRCh38.p12) following the RefSeq annotation assembly. The annotations are available from the genome database part of the NCBI database, and the data depicted above are from the Annotation Release 109 (https://www.ncbi.nlm.nih.gov/search/?term=human+genome and https://www.ncbi.nlm.nih.gov/genome/annotation_euk/Homo_sapiens/109/).

Non-coding RNA, commonly described as RNA that is not translated or does not encode for a protein, were initially considered to be non-functional [14]. However, over the years, a majority of complex organisms have shown that they transcribe ncRNAs. Many of these ncRNAs transcribed are alternatively spliced and/or processed into smaller products in the genome. A large portion of the human genome is made up of genes that do not code for any protein (Figure 2). Non-coding RNAs such as microRNAs (miRNAs), small nucleolar RNAs (snoRNAs), long non-coding RNAs (lncRNAs), circular RNAs (circRNAs), PIWI-interacting RNAs (piRNAs) and other species of regulatory ncRNAs appear to regulate signaling pathways that influence different cellular processes [15,16,17,18]. In addition, several non-coding RNAs have been identified to be associated with numerous physiological and pathological processes, leading to different diseases [17,19,20]. 

Each of these ncRNA species has been identified to regulate cellular activities such as snoRNA in RNA modification [21,22,23,24,25], piRNA on transposon silencing [26,27,28], lncRNA and circRNA in translation [29] and other regulatory functions [30], and lastly, miRNAs on gene silencing (RNA interference) [18,31] and gene regulation [32,33,34]. In spite of their known functions, ncRNAs have been described to interact with each other, forming a complex regulatory network [35]. This section will highlight some of the different ncRNA species with regard to categorization and known function. 

snoRNAs, predominantly located in the nucleolus, are ncRNAs of ~60–300 nucleotides (nt) length, and have complementarities mainly to mature ribosomal RNAs (rRNAs) involved in the post-transcription modification of ribosomal and small nuclear RNA expression [36,37]. There are two classes of snoRNAs—box C/D antisense and H/ACA snoRNAs, distinguished by their structural properties [22]. The C/D antisense box snoRNAs, which contain two short sequence motifs (box C and box D), are known to guide methylation within the pre-rRNA sequence via the regular RNA duplex formation around the methylation site [21,22]. The H/ACA snoRNAs, contain an ACA motif positioned three nucleotides from 3′ tail end and an H motif in the hinge region [23,24]. In the terminal hairpin at the 5′ and/or 3′ of the H/ACA structural internal loop, is a pseudouridylation site. This site targets uridine by base-pair interaction with rRNAs, acting as a rRNA pseudo-uridylation guide [25].

piRNAs are 24–35 nt long ncRNAs, identified mainly in the animal germline [26,38,39]. piRNAs form the piRNA-induced silencing complex (piRISC), which recognises and silences transposable elements (TEs) so as to protect the integrity of the genome [28,40]. piRNAs are generated through two distinct molecular mechanisms, primary and secondary (ping-pong) pathways [40]. The primary pathway of piRNA is an initiation phase, through which thousands of precursor/primary piRNAs displaying a bias uridine (U) are generated. These piRNAs then bind to Piwi proteins, to allow Piwi to enter the nucleus and mediate silencing [39,40,41,42]. The secondary piRNA ping-pong pathway, in *Drosophila* encoded Piwi proteins contains two main effectors—Aubergine (Aub) and Argonaute 3 (Ago3). The Aub-piRISCs initiates along with the Ago3 to produce secondary piRNAs, which cycles a ping-pong piRNA characteristic feature of 1U/10A partners and a 10-nt 5′ overlap. These two effectors act complementary to cleave sense and antisense transposon transcripts through Slicer activities silencing transposons [43,44]. In the case of the mouse piRNA pathway it is implicated in the establishment of the DNA methylation pattern essential for TE repression, while this function is apparently lacking in *Drosophila* [40,45,46].

circRNAs are ncRNAs which have 3′ head and 5′ tail ends covalently linked creating a covalently closed loop type of RNA [47,48]. Many studies have profiled circRNAs in eukaryotes (such as human [49,50,51,52] and mice [52,53]). In eukaryotic cells, circRNAs are formed through inverted splicing (/back splicing) resulting in exons of genes to attach the head to tail (forming a circRNA) [54]. circRNAs have been considered to have a potential regulatory function in translation, through acting as sponges to sequester miRNAs (~22 nt long ncRNA, which are described in more detail in the later sections) [51,55,56].

lncRNAs are in general distinguished as ncRNAs which are >200 nts long and characterized based on their location mostly encoded by intergenic regions (long intergenic/intervening (i) RNAs) and some overlapping the protein-coding genes [29,57]. lncRNAs have been categorized into different groups based on their genomic context, as-standalone, pseudogenic (promoter-associated), intronic nested antisense, terminal antisense and divergent [58]. Standalone lncRNAs are located in sequence space which do not overlap protein-coding genes in transcription, this includes some lincRNA [59]. While lncRNA, which lay intronic overlapping with natural antisense transcripts in varying degrees from none, are termed as divergent, terminal (partial overlap) and or nested (complete overlap) [58]. lncRNAs can also be pseudogenic (overlapping with pseudogenes) [60,61]. lncRNAs have been shown to be target transcriptional activators and repressors to regulate transcription [62]. Whilst post-transcriptionally, lncRNAs have been shown to be involved in pre-mRNA splicing [63,64] and translation [65]. In addition similar to circRNAs, lncRNAs alter protein translation (as well as degradation) for example through acting to sequester miRNAs from protein or mRNA targets [66,67]. lncRNAs, similar to circRNAs discussed above, have also been shown to act as miRNA sponges. A classic example is the interaction between the pseudogene *PTENP1* and its tumor suppressor parental gene *PTEN* [68]. The *PTEN* high homology 3′UTR region of *PTENP1* contains perfectly conserved seed matches for the *PTEN*-targeting miR-17, miR-21, miR-214, miR-19 and miR-26 families. These microRNAs regulate the tumor suppressor gene *PTEN*. In prostate cancer cells, the knockdown (KD) of PTENP1 leads to an increase in these microRNAs and decreased PTEN levels, resulting in increased cell proliferation [68].

### 2.2. MicroRNA Biogenesis and Function

The small endogenous miRNAs, ~22 nucleotides in length, belong to a class of non-coding RNAs first discovered in 1993 [69]. MicroRNAs predominately function as post-transcriptional gene regulators. More than 2600 mature miRNAs have been identified in humans (miRBase, version 22, accessed June 2018). It was estimated that up to 30% of protein-coding genes are regulated by miRNAs [70]. Each miRNA is predicted to have multiple potential target messenger RNAs (mRNAs) and a single gene can be modulated by several miRNAs [71], hence increasing the complexity through which miRNAs can fine-tune gene expression.

**Figure 3:**
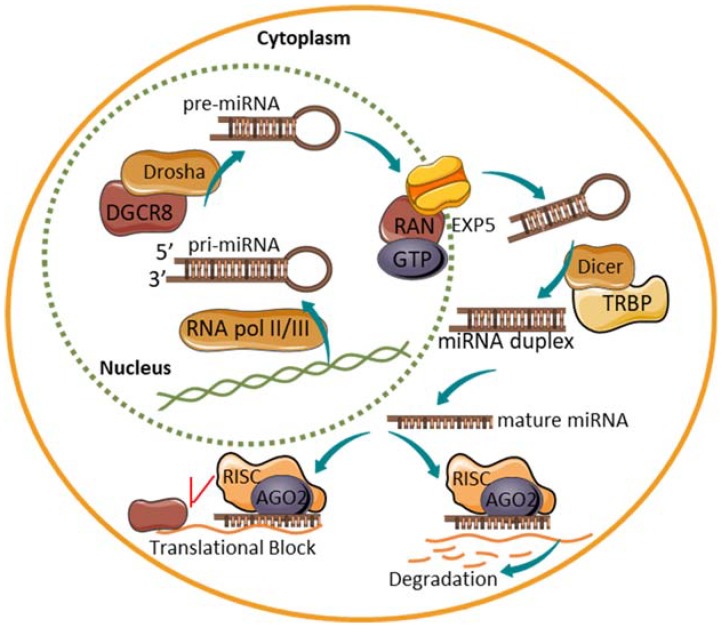
The biogenesis of miRNAs. A schematic depicting the biogenesis and function of a mature miRNA. Primary miRNA (pri-miRNA) is transcribed in the nucleus and processed by Drosha and DiGeorge syndrome critical region 8 (DGCR8). The precursor miRNA (pre-miRNA) is then exported by exportin-5 (EXP-5) out into the cytoplasm. Here, the pre-miRNA is further cleaved by Dicer to yield a double-stranded miRNA duplex. After strand selection, the mature miRNA associates with the RISC. The degree of complementarity between the miRNA and the target mRNA determines whether the mRNA is degraded or the translation process is blocked.

The canonical biogenesis and processing of miRNAs is tightly regulated at multiple stages (Figure 3). The majority of miRNA sequences are found in introns of non-coding or coding transcripts although some miRNAs are encoded within exons [72]. Some miRNAs have their own promotor, or if the miRNA resides within the introns of a protein-coding gene, the promotor of the host gene can be shared. A primary miRNA (pri-miRNA) is transcribed from miRNA genes by either RNA polymerase II, and to a lesser extent RNA polymerase III. Pri-miRNAs form an imperfect stem-loop hairpin structure with a poly(A) tail and cap typically over 1 kb in length [73]. After transcription, the pri-miRNA is endonucleolytically cleaved by the microprocessor complex comprised of the RNase III Drosha enzyme and co-factor DiGeorge syndrome critical region 8 (DGCR8), resulting in a precursor miRNA (pre-miRNA) transcript of 60–110 nucleotides in length with a short 3′ overhang [74,75,76]. Correctly processed pre-miRNA is next exported out of the nucleus upon recognition of the 3′overhang by a nuclear pore complex consisting of exportin-5 (EXP5) and GTP-binding nuclear protein RAN•GTP [77,78]. Once in the cytoplasm, another protein complex containing the enzyme Dicer further cleaves and process the RNA-duplex releasing a small double-stranded miRNA complex [79]. Subsequently, the RNA duplex is loaded onto the RNA-induced silencing complex (RISC) aided by Argonaute (AGO) 2 protein, TAR RNA-binding protein (TRBP) and protein activator of interferon-induced protein kinase EIF2AK2 (PRKRA) [80,81]. Selection of the mature single-stranded miRNA and removal of the passenger strand usually depends on the relative thermodynamic stability of the two ends of the small RNA duplex with a lower stability favoring selection [82]. Yet, the passenger strand can also be active in mRNA silencing although most often to a lesser extent than the mature major strand miRNA [83]. After strand selection, RISC in combination with the mature miRNA identifies possible target mRNA through sequence complementary of the miRNA seed sequence to the 3′ untranslated region (UTR) of the target mRNA. The seed sequence consists of 6–8 nucleotides at the 5′ end of the miRNA and it is thought that perfect complementary base pairing between the miRNA and mRNA results in a rapid degradation of the mRNA transcript. Partial complementarity between the miRNA:mRNA complex prevents the protein translation process and subsequently initiates cognate mRNA degradation. 

Although considered as highly stable cellular molecules, active miRNA degradation has been demonstrated. However, little is known of degradation dynamics, half-life and turnover of most miRNAs. The stability and turnover of selected miRNAs has been linked to specific stages in the cell cycle [84,85], growth factor signalling [86], neuronal activity [87] and highly complementary mRNA targets [88]. In contrast to mRNA, miRNAs have 5′ and 3′ unprotected ends rendering them accessible to exoribonuclease activity. Selective exonucleolytic decay of miRNAs mediated by the 3′-5′ exoribonucleases XRN-1 along with ribosomal RNA-processing protein 41 (RRP41)—a core component of the exosome complex—has been identified [89]. Without affecting pri- or pre-miRNAs, the interferon-inducible 3′-to-5′ exoribonuclease human polynucleotide phosphorylase (hPNPase (old-35)) degrades certain mature miRNAs [90]. Non-templated addition of adenosines to the 3′ end of mature mammalian miRNAs have both been observed to promote selective miRNA stability [91] and degradation [92]. Modification of the 3′ end of either pre-miRNA or mature miRNA by addition of uridines is implicated in miRNA degradation [93,94].

The recent discovery that miRNAs are intracellularly sorted and subsequently selectively secreted into the extracellular space has suggested that miRNAs participate in and are essential for cell-to-cell communication [95,96]. Encapsulated in either microvesicles (MVs) or exosomes [95,97] or bound to AGO2 [98] or lipoproteins [99,100] miRNAs can be taken up by either neighbouring or distant cells and exert their functions there. Multivesicular bodies (MVBs) are a specialised subset of endosomes containing multiple small vesicles. Exosomes (<100 nm in size) derived from MVBs are released upon MVBs’ fusion with the plasma membrane. Formed by outward budding of the plasma membrane, microvesicles (MVs) (100 nm–1 mm in size) are released into the extracellular space. The miRNA content within the exosomes does not merely reflect the miRNA composition of the parent cell suggesting that exosomal miRNA transfer is a selective process [101,102,103]. How miRNAs enter exosomes and MVs and how these processes are regulated, is not characterized.

Exosomes/microvesicles not only help the transfer of miRNAs but they also protect them from RNase degradation. Due to their high stability and resistance in degradation by endogenous nucleases, the microRNAs eventually end up at abundant levels in several body fluids, including peripheral circulation [104]. Since miRNAs are selectively secreted into exosomes, which may be recognized by their surface proteins, exosomes constitute an attractive category of biomarkers with perhaps superior tissue specific reporting [105]. Specific circulating miRNAs have been related to development of type 1 diabetes [106,107,108,109], although the identity of the miRNAs reported are not consistent between studies, more studies are needed in order to reconcile differences in findings. Nonetheless these finding have opened up a possibility of using miRNAs as predictors of future diabetes or diabetic complications, especially with the advent of newer and faster technologies to detect them with high specificity and reproducibility.

More interesting perhaps, is the report that exosomal miRNAs also confer cells with a functional mean for signal transfer to neighbouring cells and cell types. This is illustrated by the finding that lymphocytes secreted exosomes near pancreatic β-cells, which contribute to the induction of β-cell apoptosis in type 1 diabetes [110,111,112].

## 3. MicroRNAs and Non-Coding RNAs in Fetal Pancreas and Islet Development

### 3.1. Stage-Specific Expression of MicroRNAs

MicroRNAs are important at multiple stages in pancreas and β-cell development, which is demonstrated from experiments of cell and stage-specific deletion of the miRNA processing enzyme Dicer1 [113,114]. Using Pdx1 directed Cre-recombinase mediated Dicer1 deletion in mice, it was observed that pancreas development was impaired as well as β-cell mass was reduced, with the phenotype being observable before birth and mice dying early in life [115]. However, when deleting Dicer1 using a rat insulin promoter (RIP) driven Cre-recombinase, expressed specifically in the β-cells, neonatal β-cell numbers were normals, while the β-cell mass gradually reduced with age. The removal of Dicer1 in β-cells also lead to reduced insulin content, reduced insulin secretion and development of diabetes during aging [116]. Another inducible mouse model of Dicer1 deletion controlled by tamoxifen-inducible Cre-recombinase expressed only in β-cells (directed by the RIP) showed development of glucose intolerance following two weeks of tamoxifen injections, reduced insulin content and upregulation of insulin promoter transcriptional repressors [117]. This study was corroborated in another inducible Dicer1 deletion, which also showed decreased insulin secretion and exocytosis, as well as a reduced insulin content and β-cell mass. This was, furthermore, associated with the upregulation of β-cell disallowed gene transcripts [118,119]. Thus, taken together these studies firmly indicate that miRNAs, as a molecular species, are very important for early pancreas and β-cell development, as well as the function of the mature β-cell. 

Neurogenin 3 (Ngn3) is a marker of pancreatic endocrine progenitor cells [120] seen largely during the 2nd trimester and not at all in mature islet cells [33]. Although Ngn3 is necessary for β-cell formation during developmental stages, Ngn3 appears not to be expressed during pancreas regeneration induced by partial pancreatectomy. Levels of miR-15a, miR-15b, miR-16 and miR-195 were increased in mouse developing and regenerating pancreas samples following assays for 283 miRNAs, and concomitantly, these miRNAs were predicted to target Ngn3 mRNA. Thus, experiments with anti-sense treatment for miR-15a, miR-15b, miR-16 and miR-195 of single-cell suspensions from regenerating pancreas caused re-appearance of mRNAs encoding Ngn3 and its down-stream target NeuroD1 [33]. Therefore, miRNA mediated Ngn3 mRNA regulation could provide control of regeneration in the adult mouse pancreas.

In the perinatal period, a burst of β-cell replication and maturation takes place in the rat pancreas [121,122]. The miRNA profile of late developmental events in the pancreas was determined at fetal day 20, the day of birth and two days of birth. Seven miRNAs were differentially expressed perinatally and the localization studies showed endocrine localization of six of these miRNAs (miR-21, -23a, -29a, -125b-5p, -376b-3p and -451), and all were expressed in exocrine cells at one time point at least. A comparison between the mRNA and the miRNA expression pattern of perinatal pancreas showed that up-regulated miRNAs selectively target cholesterol synthetic genes, which are down-regulated following birth [123]. Interestingly, although total pancreas miR-375 levels were not regulated around birth, the localization of miR-375 changed from E20 to post-natal day 2. Interestingly, while miR-375 was detectable at robust levels at all three time points, the major site of expression of miR-375 at D0 and D2 was in pancreatic exocrine cells, while expression at e20 and in adults β-cells is endocrine cells. This indicates that pancreatic endocrine cells may not always be the major source of expression of miR-375 in pancreas and that miR-375 has a dynamic change of expression in pancreatic exocrine tissue during the perinatal period. It is suggested that the marked change of miR-375 levels in exocrine cells following birth could regulate processes involved in the adaptation of the exocrine pancreas to digestion of external nutrients derived from milk, which is lipid rich [124].

miR-7 is highly expressed both in the developing and the adult pancreas of mouse and humans [125,126,127,128]. However, miR-7 may have other roles during development compared with adult pancreas. Delivery of antisense miR-7 in vivo to mouse fetal pancreas at E10.5 cause strongly reduced insulin content at E17.5 as well as increased apoptosis throughout the pancreas. Accordingly, antisense-treated mice exhibited impaired glucose intolerance, lower insulin content and fewer β-cells [129]. These observations are contrary to the findings that inhibition of miR-7 in adult mice promotes β-cell replication [125,126]. Inhibition of miR-7a in human dispersed islets resulted in an impressive 30-fold increase in proliferation, indicating that miR-7 may serve as a negative regulator of proliferation. Moreover, the mTOR pathway is activated upon miR-7 inhibition of mouse MIN6 cells and primary mouse islets via an upregulation of 5 downstream targets of the mTOR (p70S6 K, elF4E, Mapkap1, Mknk1 and Mknk2) [126]. However, expression of miRNAs during pancreatic embryonic development has been investigated mainly using targeted micro-array methods, in which the general limitation is that only known miRNAs will be investigated.

### 3.2. lncRNAs in Human and Mouse Pancreatic Islets

The specialized function of pancreatic β-cells require massive amounts of insulin to be produced. In fact, at the transcriptome level 20% of islet mRNA reads is proinsulin mRNA (45% in β-cells) [130]. Moran et al. (2012) [130] identified 1128 transcripts as non-coding (>200 bp, no coding potential, not overlapping coding sequences. Of these, 761 were antisense, 32 had the same direction and were close to a neighboring gene transcription start, 335 were intergenic, and 55 were overlapping antisense ncRNAs to coding genes. This set of curated lncRNAs was updated to ~2400 in Akerman et al. (2017) [131]. lncRNAs generally have low expression levels (<10 RPKM), but a substantial part of the identified lncRNAs were highly islet specific. A number of these were dynamically regulated during mouse fetal pancreas development (at E15.5) or had altered levels in neonatal islets compared with adult islets (investigated as mouse orthologues) [130]. For in vitro differentiated β-like cells, patterns of dynamic regulation were observed with most of the tested lncRNAs increasing specifically in the functional endocrine differentiated cells and having low levels throughout differentiation. Human islet-long noncoding RNA (HI-LNC)-45, -60, -78 and -80 are exceptions and display expression in different stages of differentiation.

HI-LNC-67, -77 and -87 were dysregulated in ob/ob islets and HI-LNC-78 and -80 were increased by glucose treatment of human islets (4 vs. 11 mM for 72 h). Interestingly, a significant part of the islet specific lncRNAs were situated closely to genes known to have an important function in pancreatic β-cells: HI-LNC-94 (*RFX6*), -26 (*PCSK1*), -75 (*SOX9*), -5 (*ISL1*), -45 (*TUBB3*), -100 (miR-210), -101 (*PAX6*), -57 (*ISL1*), -66 (*NEUROD1*), -65 (*SIX9*), -103 (*ABCC8*). Moreover, there is an enrichment of islet lncRNAs in β-cell super enhancers, and also coincidence of the chromosomal localization of many islet lncRNAs and the presence of genetic susceptibility loci for type 2 diabetes [132,133]. 

To investigate in detail the β-cell transcriptome, Ku et al. (2012) FACS-sorted β-cells from MIP-GFP mice and sequenced poly-adenylated RNAs to first identify β-cell enriched transcripts and second to identify long ncRNAs [134]. Using this approach 1359 high-confidence lncRNA genes were identified, of which 160 showed very high β-cell specificity (>200 fold enrichment in β-cells) and 108 ncRNAs were only detected in β-cells. This number of β-cell long ncRNAs is very similar to that identified by Moran et al. (2012) [130]. Another study identified a set of 145 mouse lncRNAs using high-stringency criteria from high-purity sorted alpha and β-cells and also noted that the loci of these islet ncRNAs were highly enriched for binding sites of Pdx1, Nkx6.1, MafA and NeuroD1, as well as their expression levels being highly correlated with the expression levels of their closest neighbor genes [135]. 

Akerman et al. (2017) performed loss-of-function characterization of 12 lncRNAs by artificial microRNA mediated KD in EndoC βH1 human insulinoma cells [131]. 12 lncRNAs were selected for investigation based on the details in Moran et al. (2012) [130]; HI-LNC-12, -15, -25, -30, -70, -71, -75, -76, -78, -79, -80, -85. This selection was based on islet and β-cell enrichment, expression in EndoC cells and presence of chromatin marks indicating active promoters; moreover the 12 lncRNAs were selected due to close proximity to genes known to be important in β-cells. Working from the underlying hypothesis that lncRNAs work by modulating the transcriptome, the authors measured the transcriptomic effects of individual lncRNA KD. Here, HI-LNC-12, -15, -30, -78, -80 and -71 had measurable effects on steady-state mRNA levels in the range observed for β-cell transcription factors such as *PDX1* and *HNF1A*. HI-LNC-12, -78 and -71 KD impaired glucose stimulated insulin secretion (GSIS) and insulin content of EndoC cells and many of these lncRNAs had expression levels that were highly correlated with each other and with β-cell transcription factors (*GLIS3*, *HNF1A*, *NKX2.2*, *PDX1*, *MAFB*). In particular, HI-LNC-78 correlated with these transcription factors, while HI-LNC-25 did not [131]. 

The tested HI-LNCs also regulated genes in pathways controlling insulin secretion and regulated a largely overlapping gene set as these β-cell transcription factors. HI-LNCs more often than by chance regulated genes associated with enhancer clusters of islet chromatin. Gene-module co-expression analysis of human islet RNA-seq data indicated that HI-LNC-12, -15, -71, -78, and -80 were part of a transcriptional regulatory network with the transcription factors investigated (*MAFB*, *GLIS3*, *HNF1A*, *PDX1*, *NKX2.2*). They then proceeded to functionally investigate the specific functional effects of HI-LNC-71 (which they termed *PLUTO* for *PDX1* locus upstream transcript one) on PDX1 levels. The KD of *PLUTO* decreased *PDX1* mRNA levels in EndoC cells and dispersed human islet cells, while the converse was not observed. Down-regulation of *PLUTO* using CRISPRi also decreased *PDX1* levels and the regulome of *PLUTO* was highly overlapping that of *PDX1*. Mechanistically, *PLUTO* transcript KD decreased the physical association of *PDX1* enhancers with the *PDX1* proximal promoter and transcription initiation site. Thus, it was concluded that *PLUTO* acts as a scaffold to assist the formation of a tight chromatin structural assembly on the *PDX1* promoter [131]. It will be interesting to learn which parts of the *PLUTO* transcript that mediates this scaffolding effect and whether *PLUTO* also assists as a scaffolding RNA in other β-cell enhancer complexes. 

Mouse βlinc1 is the syntenic orthologue of HI-LNC15, which was demonstrated to control NKX2.2 levels in EndoC cells and whose transcript also correlated significantly with *NKX2.2* mRNA levels [136]. HI-LNC-15 was also part of the same network of β-cell transcription factors as HI-LNC-12, -71, -78, and -80 [131]. βlinc1 is a 6.8-kb spliced non-coding transcript located in a region of open chromatin ∼20 kb upstream of *Nkx2.2*. Similar to HI-LNC-15, βLINC1 is enriched in islet tissue, and it is localized to the nucleus. SiRNA mediated down-regulation of βlinc1 in MIN6 cells decreased in *Nkx2.2* mRNA levels. Removal of βlinc1 by knock-out (KO) in mice resulted in mild glucose intolerance, a lower number of insulin positive β-cells and a markedly increased amount of somatostatin positive δ-cells. The expression of βlinc1 in the fetal stages and the altered transcriptional program observed in βlinc1 KO pancreata supports a role in the correct specification of endocrine precursor cells during the secondary transition of the pancreas [136], which is highly important for β-cell differentiation (Figure 1). Moreover, the regulatory potential of βlinc1 was highly associated with neighboring loci, suggesting that this ncRNA acts mainly in cis. 

Mouse βlinc2 and -3 were also recently described [137]. βlinc2 is upregulated in high-fat diet responders and in db/db, and correlated positively with increased weight and glycemia, although moderately with insulin levels, while the opposite was observed for βlinc3. Both lncRNAs were enriched in islets, and especially in β-cells. βlinc2 was increased in islets by glucose culture and palmitate treatment, while βlinc3 was not regulated by glucose and decreased by palmitate. Human βLINC 3 was similarly regulated as βlinc3 and decreased in type 2 diabetes (T2D), and human islet expression levels of βLINC 3 correlated negatively with BMI and HbA1c of the donors. Functional investigations of βlinc2 and -3 in mouse MIN6B cells indicated that over-expression of βlinc2 increased apoptosis, while depletion of βlinc3 increased apoptosis. Thus, due to their regulation by nutrients and effects on pancreatic β-cells these lncRNAs are good candidates for having a role in development tof β-cell dysfunction and T2D. However, it is not possible to easily identify the ncRNAs in the Genome Browser (https://genome.ucsc.edu/) based on their chromosomal localizations, as these lncRNAs are not annotated. This highlight an issue with ncRNA research in the sense that it can pose challenges for replication of findings by others, if databases, such as Genome Browser or lncrnadb.org are not continuously updated with these results. 

### 3.3. piRNAs in Pancreatic Islets

Piwi interacting RNAs (piRNAs) are present in pancreatic islets and β-cells [138]. Comparing piRNA expression of rat isolated islets from adult and P10 animals revealed multiple piRNA species that were markedly decreased in adult rat islets. In islets from GK rats, DQ751874 was decreased, while DQ746748 and DQ732700 piRNAs were increased compared with control islets. RNAi mediated depletion of *Piwi2* and *Piwi4* mRNAs, also decreased the level of these piRNAs. Further studies investigating functional impact of *Piwi2* and *Piwi4* depletion in dispersed rat islet cells showed that GSIS was decreased and that these protected against cytokine-induced apoptosis, while proliferation was unaffected. Over-expression of DQ746748 and DQ732700 piRNAs also had no impact on apoptosis or proliferation but decreased GSIS, while the mechanisms through which this happens were not characterized. Published studies of piRNA mediated effects in pancreatic β-cells are very rare, and seeing as these are both dynamically regulated in pancreatic islets or β-cells and have functional effects on insulin release, it will be relevant to study this category of ncRNAs further.

### 3.4. Pancreatic Islet Circular RNAs

Stoll et al. (2018) identified 3441 circular RNAs as being expressed in human islet, and 497 of them had exact orthologues expressed in mouse islets [139]. The ciRS-7 (CDR1as), originally described in brain tissue [55], is also expressed in pancreatic β-cells, where its over-expression upregulates islet cell insulin secretion and insulin content [140] and KD reduces insulin secretion, insulin content and decreases prolactin induced proliferation of MIN6B cells and isolated rat islets [139] by, at least in part, binding available to miR-7. The most abundant islet circular RNA species is circHIPK3, which was decreased in db/db islets. Moreover, depletion of circHIPK3 resulted in increased apoptosis, decreased proliferation and GSIS. CircHIPK3 depletion caused a decrease in the expression of a number of β-cell genes involved in insulin secretion and also decreased the levels of the linear HIPK3 transcript [139]. As reported for other circular RNAs, circHIPK3 appeared to act in part by microRNA sponging of miR-124-3p, miR-29-3p, miR-338-3p and miR-30, all of which are miRNAs which may play a role in either β-cell development, function, proliferation or survival [113,141,142,143,144,145]. Other circRNAs, such as *MAN1A2*, *RHOBTB3* and *RMST* are also highly expressed in pancreatic islets, while the circRNA derived from TGFBR3 was highly β-cell selective, and the cirRNAs from *FAP*, *SYTL5*, *PTPRT*, *STK32B*, and *BVES* were highly alpha cell selective [146].

### 3.5. NcRNAs in Pancreatic Alpha Cells versus β-Cells

Since more research tools and models exist for the study of pancreatic β-cells, more knowledge regarding miRNAs has been accumulated on β-cells than for other islet cell types. Thus, expression studies have generally been performed in isolated islets, followed up with functional studies in dispersed islet cells or insulin-secreting cell lines such as MIN6 and INS-1 832/13. The differential miRNA expression in islets from T2D human donors, and/or diabetic animal models, has been investigated in several studies and has recently been reviewed [147]. 

A few studies have investigated cell sorted mature α- and β-islet cell fractions. An RNA sequencing study of sorted human alpha and β-cells revealed that 328 of the 384 unique miRNAs were shared [148]. miR-375, miR-7-5p, miR-148a-3p, miR-26a-5p, miR-127-3p, miR-27b-3p, miR-192-5p, miR-143-3p and the let-7 family were among the most highly expressed miRNAs in whole islets and isolated β-cells. Another study, based on a polymerase chain reaction (PCR)-based miRNA array platform, identified 141 miRNAs as being expressed, in which 134 were preferentially expressed in β-cells whereas only 7 were more expressed in alpha cells [149]. Clearly, more knowledge about the role of miRNAs and other non-coding RNAs in alpha cells are needed, however, research in this area is severely hampered by the lack of widely available cell line models for alpha cells [113]. 

### 3.6. Imprinted ncRNAs and β-Cell Development

**Figure 4:**
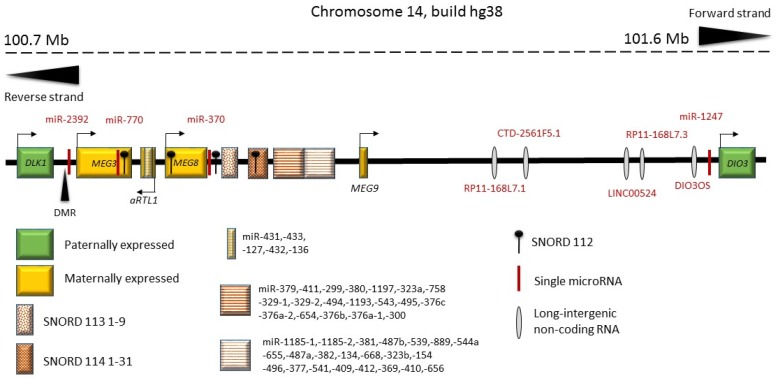
An overview of the imprinted genomic region on human chromosome 14 between *DLK1* and *DIO3*. Genes marked in green are paternally expressed and genes marked in orange are maternally expressed. DMR: the *MEG3* differentially methylated region. Shown are also single miRNAs, the miRNA clusters, *SNORD* genes and other ncRNAs. Redrawn and updated with inspiration from Benetatos et al. (2013) [157].

β-Cell dedifferentiation occurs in human T2D as well as in mouse [150,151]. Using small RNA sequencing to identify non-coding RNAs altered in human T2D, Kameswaran et al. (2014) investigated a cluster of miRNAs that were consistently decreased in type 2 diabetic islets [152,153]. Interestingly, these miRNAs mapped to a common region on chromosome 14q32, which is known to be imprinted [153,154] (Figure 4). Genomic imprinting is an epigenetic mechanism that results in only one of the two parental alleles being expressed. Only about 100 human genes are imprinted with most of these being conserved between humans and rodent. Imprinted loci contain parental allele-specific differences in DNA methylation patterns at specific imprinting control regions (ICRs). These differentially methylated regions (DMRs) are known to regulate the expression of imprinted genes. Imprinted genomic regions often contain non-coding RNAs—both lncRNAs and miRNAs—which have been demonstrated to play a role in the maintenance of the imprinted state of the region [155]. The imprinted human chromosome 14q32 (chromosome 12 in mice, chromosome 6 in rats) region is flanked by the genes delta-like homolog 1 (*DLK1*) and type 3 iodothyronine deiodinase 3 (*DIO3*), which are paternally expressed (Figure 4). Within the *DLK1-DIO3* region, there is a number of ncRNAs: *MEG3* (called *Gtl2* in mouse) [156], *MEG8* (called *Bsr* in rat and *Rian* in mouse), and antisense RTL1, C/D snoRNAs and a very large miRNA cluster [157]. This miRNA cluster is currently the largest known in the human genome and contains 53 miRNAs on the forward strand and 1 on the reverse strand. 

The T2D down-regulated miRNAs in the *DLK1-DIO3* region are generally very highly expressed in islets and enriched in β-cells [152]. Moreover, this cluster is also down-regulated in rat newborn pancreas following maternal gestational obesity [158]. *Bsr* (the rat ncRNA homolog of human *MEG8*) was also suppressed by gestational obesity, and in vitro studies of isolated neonatal rat islets exposed to cytotoxic cytokines showed that these suppressed the expression levels of *Bsr* in a dose and time-dependent fashion [158]. Functional target RNA identification using the HITS-CLIP method identified the *TP53INP1* mRNA as being a target of miR-495, one of the T2D and gestational obesity suppressed miRNAs [152,158]. The protein TP53INP1 is induced by p53, plays a role in β-cell stress response and is located in the genetic T2D susceptibility region [159]. The *MEG3* DMR of the *DLK1-DIO3* imprinted region shows increased methylation levels in human T2D islets [152]. Targeted methylation of the promoter of *MEG3* in the DMR, resulted in a decreased transcript of the maternal transcripts in this region. Moreover, this was accompanied by an increased sensitivity to cytotoxic cytokines and decreased binding of β-cell transcription factors [160]. Thus, the transcriptional activity of the imprinted DLK1-DIO3 region is suppressed in T2D and by fetal-maternal programming.

*Meg3* (mouse *Gtl2*) has been shown to affect insulin biosynthesis and secretion, promote apoptosis and decrease β-cell maturation [161] and is also differentially expressed during embryonic development [156]. Interestingly, this imprinted *Dlk1-Meg3* region, while being important for β-cell function and development, also constitutes a Type 1 diabetes susceptibility locus [162]

Box C/D snoRNAs participate in the post-transcriptional modification of ribosomal RNAs and mRNAs. *Bsr*/*MEG8* contains C/D snoRNAs in 86 tandem repeats [158] (Figure 4). Another imprinted locus, on human chromosome 15q11-q13 (Prader Willi syndrome region) also contains a large number of tandemly repeated C/D snoRNAs. The link between genomic imprinting and tandemly repeated C/D snoRNAs suggest a role for them and their host non-coding genes in the mechanism of the epigenetic imprinting process [163].

Although PWS is mostly caused by a large 5 Mb deletion of the entire PWS gene region on chromosome 15, the identification of patients harboring smaller microdeletions has enabled the definition of a 91 kb PWS minimum critical deletion region, which contains only three ncRNAs; a lncRNA named IPW, a single copy snoRNA, SNORD109A and a snoRNA cluster of 30 repeated units; SNORD116. The role of C/D box snoRNAs is usually to methylate nucleolar rRNAs prior to their nuclear export [37], however this role has not been confirmed for SNORD116 as no specific rRNA target of this snoRNA has been identified. Interestingly, PWS individuals display relative hypoinsulinemia, suggesting a role for these three ncRNAs in β-cell development and function. Investigation of a mouse model of paternal allele deletion of *SNORD116* (*Snord116^p−/m+^*) showed decreased islet size of neonatal and adult mice. *Snord116^p−/m+^* islets were found to have unaltered numbers of insulin positive cells, but decreased numbers of glucagon positive cells and increased numbers of somatostatin positive cells. While the number of β-cells appeared normal, the level in insulin (*Ins2*) mRNA transcripts was markedly decreased [164]. Moreover, *Snord116^p−/m+^* islets were found to contain increased amounts of polyhormonal cells at birth indicating a state of impaired endocrine cellular differentiation. These findings suggest that the *SNORD116* cluster is important for fetal β-cell differentiation

*H19* is a maternally imprinted intergenic lncRNA generated from the *Igf2* locus, is highly expressed in neonatal islets and completely suppressed in adult islets (rat), declining from P1 to P31 [165]. This expression is dependent on the presence of E2F1 as siRNA mediated KD of E2F1 decreases *H19* levels. *H19* increases and is necessary for β-cell proliferation, but does not protect against cytokine-induced apoptosis and no change in GSIS or insulin content was observed following over-expression. While the RISC partner Ago2 was required for the proliferative effect of H19, H19 overexpression specifically led to over-expression of miR-675-5p and depletion to reduced amounts of miR-675-5p in dissociated islet cells. Although miR-675-5p (and -3p) levels declined from P1 to P31 in a similar fashion as *H19*, the effect of *H19* on β-cell proliferation was not dependent on miR-675 species as anti-miR-675-5p or -3p did not affect β-cell proliferation. However, *H19* also contains let-7 binding sites and over-expression of a *H19* variant without these binding sites (*H19*Δ) did not cause β-cell proliferation in adult dissociated islet cells or rat insulinoma cells [165]. Moreover, antagonizing all let-7 family members in adult rat islets increased β-cell proliferation. *H19* over-expression also increased Akt phosphorylation, which was dependent on the presence of let-7 binding sites, and Akt phosphorylation and PI3 kinase activation was required for the β-cell proliferative effect of *H19*. 

*H19* is part of the imprinted *IGF2* region, but regulating **H19** levels by over-expression or siRNA mediated degradation did not alter *IGF2* mRNA levels. Thus, at the molecular level, the *H19* ncRNA generates miR-675-5p and -3p, but this mechanism is not part of the β-cell proliferative effect of *H19* and these current results demonstrate that the let-7 binding or depleting actions of *H19* are involved in β-cell proliferation, which along the way involves Akt phosphorylation and PI3 kinase activity. 

Thus, the possible role of imprinted regions for β-cell development is further emphasized as these are involved in regulating ncRNAs in pancreatic β-cells, are regulated in T2D and by fetal gestational programming and are linked genetically to T2D and β-cell differentiation. However, to clarify the roles of these regions and their ncRNAs sufficiently, more in depth functional studies are needed of their impact on pancreas and β-cell development as well as β-cell survival and proliferation.

### 3.7. Fetal Programming and ncRNAs in Control of β-Cell Growth and Exocytosis

The functional capacity of the endocrine pancreas, β-cell numbers, and volume, is decreased by intrauterine fetal programming as it occurs following malnutrition such as low-protein malnutrition or caloric restriction during late gestation. Moreover, reduced β-cell mass in conjunction with lower peripheral insulin sensitivity increases the risk of developing type 2 diabetes in adulthood [122,166,167]. 

In rat and mouse islets, which display similar phenotypes following the maternal gestational low-protein diet, quite different miRNA profiles were observed [168,169]. While in rats at age 3 weeks, differentially regulated miRNAs were in general upregulated (i.e., miR-375, miR-30 and miR-200 members) [168,170], mouse islets showed a majority of down-regulated miRNAs, one of these being miR-30b, while only miR-7, miR-99, and miR-199 were up-regulated [169]. The observed differences, could either be due to species differences or be due to the different time points of investigation (3 weeks versus 12 weeks).

mTOR signaling is reduced in gestational low-protein diet treated offspring mice, and the islet miRNA profile showed enrichment for targets in mTOR signaling, which was followed up by target validation that miR-7 targets the mTOR pathway in pancreatic islets inhibiting β-cell proliferation in vitro. MiR-7 family members, miR-99, mir-152, miR-199, and miR-342 were increased in islets from 3-month-old mice, and inhibiting miR-199 and miR-342 increased mTOR protein levels [126]. Thus, miRNAs of the highly expressed miR-7 family mediate impaired β-cell proliferation in response to gestational low-protein diet by inhibiting the mTOR pathway. Interestingly, the fetal expression of miR-7 is detected from E3.5 with a peak expression level at E14.5–E16.5, after which expression levels wane. Moreover, as mentioned above, inhibiting miR-7 in vivo in the fetus using anti-sense oligos decreased the number of β-cells, lowered insulin content and impaired glucose tolerance in adult mice [129]. Transgenic mouse models also support that miR-7 family members are important for the proper mature β-cell function. Thus, RIP-directed miR-7a over-expression in β-cells leads to diabetes via β-cell dedifferentiation and impaired insulin secretion, while β-cell miR-7 KO increased GSIS and insulin exocytosis [171]. Of note, in this model, miR-7 alterations did not affect β-cell mass or islet organization, indicating that while miR-7 may be important for β-cell mass via its fetal expression, its role in mature β-cells appears to be control of insulin exocytosis. 

miR-375 was increased by a gestational low-protein diet, and over-expression of miR-375 in rat primary dissociated islets cells caused impaired islet cell proliferation and decreased insulin secretion. Conversely, inhibition of miR-375 reversed these phenotypes [168]. Of note, when miR-375 was over-expressed in human islets cells following in vitro expansion, it enhanced the directed re-differentiation into β-cells, increased insulin content and markers of mature β-cells, but also caused a decrease in β-cell replication [172]. Interestingly, in vitro expansion and dedifferentiation also resulted in decreased expression of miR-7, miR-335, miR-30 and miR-200 family members as well. Thus, these results indicate that premature expression or up-regulation of miR-375 promote the induction of a more mature β-cell phenotype and simultaneously reduce β-cell proliferation and, therefore, total β-cell numbers, possibly acting as a switch mechanism. This may be due to interactions with targets such as *PDPK1* (3′-phosphoinositide dependent protein kinase 1); an upstream regulator of Akt and GSK3 activity [168,172,173]. Moreover, the miR-375 global KO mouse model not only displays decreased insulin exocytosis but also has decreased β-cell mass and β-cell numbers [174,175]. Thus, it is possible that miR-375 mainly controls β-cell proliferation during fetal development, while it controls insulin exocytosis in the mature β-cell. Of note, miR-375 does not seem to be regulated during aging (newborn, young, adult, old age rodents) [123,176,177], although it may change cellular expression patterns at different time points [124]. 

### 3.8. Species Differences in ncRNAs between Mouse and Human

Although mouse and human pancreas and β-cell development is very similar, there are some notable differences. The differentiated adult human islet contains relatively more alpha cells than rodent islets, and is structurally less organized [178]. During development of the human pancreatic islet, there is an initial organization (week 14) of the endocrine cells in clusters, in which β-cells form a core with a periphery of alpha cells and delta cells similar to mouse. Later in week 18, the peripheral alpha cells and delta cells migrate from the β-cell core to form homogenous juxtaposed islets. The homogenous islets would reintegrate and form mature islets after week 22 that resemble adult islets [179].

Reflecting differences in pancreatic islet development, it is worth considering between species conservation of ncRNAs as a source of these, as also reviewed recently [114]. While Moran et al. (2012) only investigates human cells, it has been observed that one third of the identified lncRNAs do not have mouse orthologues [114,130], and whereas only one out of seven circRNAs were species conserved [139]. The very low species conservation rate for lncRNAs underlines the observation that very few lncRNAs act by extensive base pairing, but rather as scaffolds or chromatin-structuring entities, which does not enforce a high degree of nucleotide conservation. 

Several lncRNAs such as *XIST* [180], *NEAT1* [181,182], or the cardiac mesoderm enhancer-associated non-coding RNA (*CARMEN*) [183], have been shown to have conserved loss-of-function phenotypes across species. However, while *HOTAIR*, a lncRNA controlled the expression of the *HOXD* (homeobox D) cluster in primary human fibroblasts [184], the murine counterpart did not regulate the *HoxD* cluster [185]. In cases wherein lncRNAs retain functionality across species, it remains unclear if they act through the same targets. A previous study [186] demonstrated that although the functionality of the lncRNAs is conserved, their binding sites/mechanism of action could be drastically different across species. The important need of current time is, therefore, to improve the depth and coverage of sequencing data across all species and the availability of better computational tools to identify short stretches of conservation across species. 

The majority of human miRNA families are well conserved in the mouse. Currently, miRBase counts 2654 human and 1978 mouse mature miRNAs. However, although miRNAs are very conserved, their target mRNAs are not conserved to the same degree. Numerous examples exist for which pathways targeted by miRNAs may be evolutionarily conserved, but the individual target mRNAs are not [113,123]. This underscores the need for investigation of miRNAs in the proper species context, as in the example of islet expressed miR-206, which targets murine but not the human glucokinase [187]. Thus, species differences in islet development and function may in part arise by non-conservation of ncRNAs and their targets. 

## 4. Discussion and Conclusions

Research in islet and β-cell development and function is highly relevant in respect of finding a cure for diabetes mellitus. Technological revolution in RNA-sequencing workflows and single cell transcriptome analyses have demonstrated the inherent heterogeneity that exists in biological systems. Considering the cell-/tissue-specific nature of several ncRNAs [58,168,169], it is very likely that the number of ncRNAs that make up the major classes (e.g., miRNAs, lncRNAs) would exceed the number of protein-coding genes that have been identified in the human genome. The discovery of ncRNAs that are directly linked to survival and function of insulin-producing cells will be critical in understanding their potential in predicting, preventing and/or reversing diabetes. The loss of β-cell mass associated with both type 1 diabetes (T1D) and T2D has attracted efforts to identify alternative routes to islet transplantation, such as that of endogenous pancreatic β-cell regeneration as seen in rodent under both non-diabetic as well as diabetic conditions [188,189]. In humans, the proliferation of adult pancreatic β-cells is low to undeterminable under steady-state conditions [190,191,192] although generation of new β-cells either from replication or via neogenesis happens during pregnancy [122,193]. Finding novel mechanisms involved in β-cell development and regeneration could provide new paths to increase the number of functional β-cells in patients with diabetes or aid in generating functionally mature β-cells for transplantation using in vitro protocols. As the field continues to add in more information on ncRNAs related to islet cell survival/function, approaches spanning multiple disciplines would be necessary. Given that the majority of the diabetes-associated nucleotide variants are located in the non-coding gene regions, integrated approaches to understanding the impact of such genomic polymorphisms on ncRNA stability and ultimately on the translation of islet cell transcripts, would help in understanding the role of ncRNAs in pancreatic β-cell development and function.

The research field of non-coding RNA is rapidly expanding and is an area of intense research activity. This is also the case with regard to ncRNAs in islet and β-cell function and development. Mounting evidence points to important roles of β-cell-specific lncRNAs in regulating the chromatin structure of active enhancers to maintain the appropriate gene expression patterns of highly differentiated β-cells, and in this way support the highly differentiated stage of mature β-cells, such as in the example of *PLUTO*. However, lncRNAs may not only work as scaffolds but also as sponges for expressed miRNAs or as host-transcripts from which miRNAs may be excised, such as for some of the lncRNAs for imprinted regions. To add to the complexity of ncRNAs in cellular biology, it appears that there is a large degree of interaction with regard to functional effects of different classes of ncRNAs, since for example circRNAs as well as lncRNAs may act as miRNA sponges, and piRNAs may sometimes enter the RISC and function as miRNAs. Some lncRNAs are at the same time host genes for miRNAs and may also have additional effects, for example as a scaffold and as a sponge, as in the example of *H19*, which is the host gene of one miRNA and acts as a sponge for another miRNA.

Thus, although much knowledge is accumulated, a large task of functionally verifying the actions of different ncRNAs in β-cell development and β-cell function remains. Moreover, since ncRNAs tend to have very cell-specific expression profiles, there is also a large task remaining of identifying ncRNAs in the minor cell populations of the islet and during fetal pancreas and islet development.
